# (3S*,4S*,*E*)-*tert*-Butyl 3,4-dibromo-5-oxo­cyclo­oct-1-ene­carboxyl­ate

**DOI:** 10.1107/S1600536811053852

**Published:** 2011-12-23

**Authors:** Magda Blanco, Narciso M. Garrido, Francisca Sanz, David Diez

**Affiliations:** a Departamento de Quimica Organica, Universidad de Salamanca, Plaza de los Caidos, 37008 Salamanca, Spain; b Servicio General de Rayos X, Universidad de Salamanca, Plaza de los Caidos, 37008 Salamanca, Spain

## Abstract

The title compound, C_13_H_18_Br_2_O_3_, was prepared by a bromination reaction of (1*E*,3*Z*)-methyl 5-oxocyclo­octa-1,3-diene­carboxyl­ate, which was obtained by an ep­oxy­dation reaction of *tert*-butyl cyclo­oct-1,3-diene­carboxyl­ate. The crystal structure confirms unequivocally the absolute configuration of both chiral centres to be *S*. In the crystal, C—H⋯O inter­actions link the mol­ecules into chains running along the *c* axis.

## Related literature

For the Michael addition of enanti­omerically pure lithium amides, see: Davies *et al.* (2005 ▶). For their importance in pharmacology, see: Fülöp *et al.* (2001 ▶). For the reactivity of the cyclo­octa-1,5-diene in basic medium, see: Huber *et al.* (1969 ▶, 1970 ▶). For the preparation of analogous unsaturated cyclo­octane esters, see: Garrido *et al.* (2008 ▶).
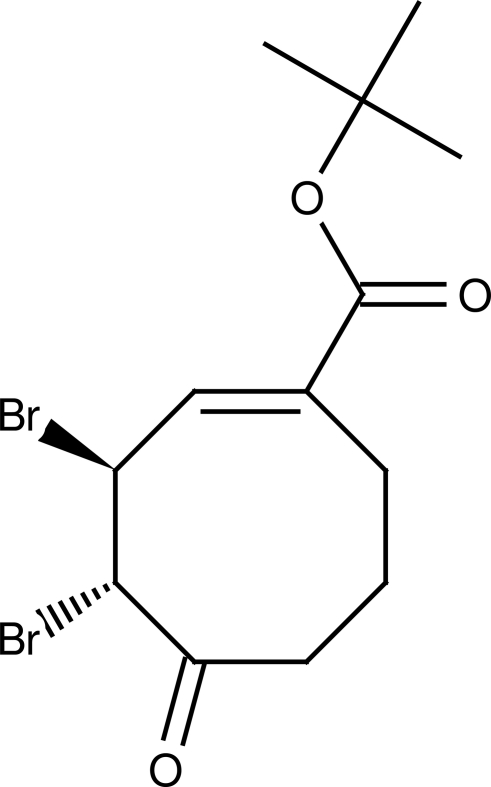


## Experimental

### 

#### Crystal data

C_13_H_18_Br_2_O_3_
*M* *_r_* = 382.09
Orthorhombic, 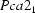

*a* = 14.0658 (4) Å
*b* = 9.5990 (3) Å
*c* = 11.2657 (3) Å
*V* = 1521.07 (8) Å^3^
*Z* = 4
Cu *K*α radiation
μ = 6.76 mm^−1^
*T* = 298 K
0.24 × 0.14 × 0.10 mm

#### Data collection

Bruker APEXII CCD area-detector diffractometer
Absorption correction: multi-scan (*SADABS*; Bruker, 2006 ▶) *T*_min_ = 0.370, *T*_max_ = 0.509
10215 measured reflections
2170 independent reflections
2153 reflections with *I* > 2σ(*I*)
*R*_int_ = 0.048

#### Refinement

*R*[*F* ^2^ > 2σ(*F* ^2^)] = 0.029
*wR*(*F* ^2^) = 0.075
*S* = 1.09
2170 reflections
166 parameters
1 restraint
H-atom parameters constrained
Δρ_max_ = 0.32 e Å^−3^
Δρ_min_ = −0.46 e Å^−3^
Absolute structure: Flack (1983 ▶), 803 Friedel pairs
Flack parameter: 0.06 (3)

### 

Data collection: *APEX2* (Bruker 2006 ▶); cell refinement: *SAINT* (Bruker 2006 ▶); data reduction: *SAINT*; program(s) used to solve structure: *SHELXS97* (Sheldrick, 2008 ▶); program(s) used to refine structure: *SHELXL97* (Sheldrick, 2008 ▶); molecular graphics: *Mercury* (Macrae *et al.*, 2006 ▶); software used to prepare material for publication: *SHELXTL* (Sheldrick, 2008 ▶).

## Supplementary Material

Crystal structure: contains datablock(s) global, I. DOI: 10.1107/S1600536811053852/bt5753sup1.cif

Structure factors: contains datablock(s) I. DOI: 10.1107/S1600536811053852/bt5753Isup2.hkl

Supplementary material file. DOI: 10.1107/S1600536811053852/bt5753Isup3.cml

Additional supplementary materials:  crystallographic information; 3D view; checkCIF report

## Figures and Tables

**Table 1:** Hydrogen-bond geometry (Å, °)

*D*—H⋯*A*	*D*—H	H⋯*A*	*D*⋯*A*	*D*—H⋯*A*
C3—H3⋯O3^i^	0.98	2.57	3.525 (5)	165
C8—H8*A*⋯O3^i^	0.97	2.63	3.590 (6)	172

Symmetry code: (i) 
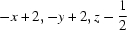
.

**Fig. 1.**
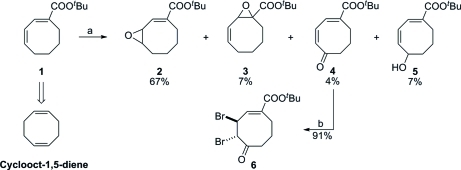
Reaction scheme for the synthesis of the title compound.

**Fig. 2.**
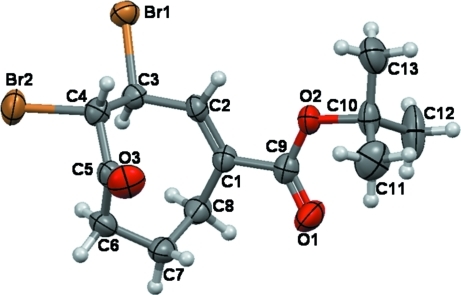
Molecular structure of C13H18Br2O3. Displacement ellipsoids are drawn at the 50% probability level. Hydrogen atoms are shown as spheres of arbitrary radius.

**Fig. 3.**
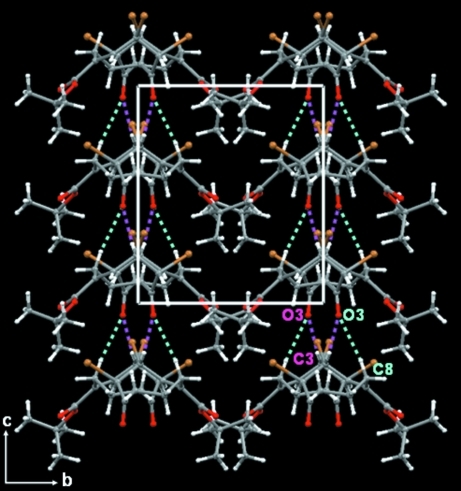
Crystal packing of C13H18Br2O3 view along a axis, showing intermolecular C—H···O interactions.

## supplementary crystallographic information

### Comment

In our research group there has been an enormous interest in the synthesis of conjugated unsaturated esters used as starting material in the Michael addition of enantiomerically pure lithium amides (Davies *et al.*, 2005) as a base tool in the asymmetric synthesis of β-amino acids and alkaloids because of their interest and value in the development of biologically active compounds for the pharmacology industry (Fülöp *et al.*, 2001). Considering that the reactivity of the cycloocta-1,5-diene is very peculiar, highlighting its trend in basic medium to conjugate its double bonds (Huber *et al.*, 1969) because of the greater thermodynamic stability (Huber *et al.*, 1970), is necessary and important to establish the exact structure in this class of unsaturated rings. This conjugation was determined previously for an isomer of compound 1 (*tert*-butyl cyclooct-1,7-dienecarboxylate) (Garrido *et al.*, 2008) and herein for compound 1 (*tert*-butylcyclooct-1,3-dienecarboxylate) by R—*X* spectroscopy of compound 6 which confirms unequivocally its configuration and structure. The crystal was afforded by epoxydation reaction of compound 1 with MCPBA and bromination reaction of compound 4 (Fig. 1).

The crystal contains an unique molecule in the asymmetric unit. The title molecule consists of a ring cyclooctene with two bromine atoms, a carbonyl group and a *tert*-butoxycarbonyl group as susbtituents. All the bond lengths and angles are within the normal ranges. The Br1—C3 and Br2—C4 bond lengths are 1.956 (4) Å and 1.946 (4) Å, respectively. The bromine atoms at C3 and C4 are nearly coplanar with the cycloctene ring being the Br1—C3—C4—C5 and Br2—C4—C3—C2 torsion angles of 173.6 (3)° and -173.0 (1)°, respectively. In the case of the *tert*-butoxycarbonyl group at C1 is also coplanar with the cycloctene ring being the O2—C9—O1—C1 torsion angle of 178.2 (7)°. The carbonyl group at atom C5 is twisted with the cycloctene ring being the O3—C5—C4—C3 torsion angle of 123.3 (8)°.

In the crystal structure, molecules are connected by intermolecular C—H···O interactions to form infinite chains running along [001] direction, which seems to be effective in the stabilization of the structure (Table 1).

### Experimental

*Epoxydation reaction, synthesis of (1E,3Z)-tert-butyl 5-oxocycloocta-1,3-dienecarboxylate*4. Compound 1 (623.8 mg, 3.0 mmol, 1 equiv) was dissolved in DCM (30 ml), and stirred at 0°C, MCPBA (568.5 mg, 3.3 mmol, 1.1 equiv) was added slowly and the solution was stirred for 5 h at room temperature. The reaction mixture was quenched with saturated Na_2_S_2_O_3_ (10 ml), extracted with DCM (3 *x* 80 ml), washed with H_2_O, saturated NaHCO_3_ and Na_2_S_2_O_3_. The combined organic extracts were dried over Na_2_SO_4_, filtered and concentrated *in vacuo*. Purification by silica gel for flash column chromatography (Hex/EtOAc (99:1 *v*/*v*) gave recovery of starting material (16%), (1*Z*,3*R**,4*S**) *tert*-butyl cycloocta-1,3-diene carboxylate 3,4 oxide 2 (450 mg, 67%), (1*R**,2*S**,3*E*) *tert*-butyl cycloocta-3,4-diene carboxylate 1,2 oxide 3 (47 mg, 7%), (1E,3*Z*)-*tert*-butyl 5-hydroxycycloocta-1,3-dienecarboxylate 5 (47 mg, 7%) and (1*E*,3*Z*)-*tert*-butyl 5-oxocycloocta-1,3-dienecarboxylate 4 as a pale yellow oil (27 mg, 4%), IR νmax (neat): 2976 and 2868 (C—H), 1707 (C=OOtBu), 1663 (C=O), 1456, 1370, 1292 (C—O), 1252, 1157 cm^-1. 1^H NMR (400 MHz; CDCl_3_): δ 1.52 (9*H*, s, COOC(CH_3_)_3_); 2.10 (2*H*, quint, J 6.6, H-7); 2.50 (2*H*, t, J 6.6, H-8); 2.57 (2*H*, t, J 6.6, H-6); 6.03 (1*H*, d, J 12.6, H-4); 6.57 (1*H*, dd, J 5.5 and 12.6, H-3); 7.26 (1*H*, d, J 5.5, H-2). ^13^C RMN (50 MHz; CDCl_3_): δ 26.3 (CH_2_, C-7); 28.0 (CH_3_*x* 3, COOC(CH_3_)_3_); 31.8 (CH_2_, C-8); 38.5 (CH_2_, C-6); 81.4 (C, COOC(CH_3_)_3_); 133.6 (CH, C-4); 134.7 (CH, C-2); 135.8 (CH, C-3); 140.1 (C, C-1); 165.5 (C, COOC(CH_3_)_3_); 205.2 (C, C-5). m/*z* (Cl^+^) (*rel*. intensity): 222 (*M*^+^, 5) 205 (3), 186 (5), 166 (19), 149 (19), 121 (22), 94 (13), 77 (26), 57 (100).

*Synthesis of (3R*,4R*,E)-tert-butyl 3,4-dibromo-5-oxocycloocta-1-enecarboxylate*6. Compound 4 (27.00 mg, 0.12 mmol) was dissolved in CCl_4_ (10 ml) and the reaction system was stirred and cooled down at 0°C. After, Br_2_ (0.01 ml, 31 mmol) was added and stirred for 30 min, the ice bath was removed and stirred for 4 h at r.t. The reaction mixture was dissolved in DCM (20 ml), washed with HCl 2 N., NaHCO_3_(sat.), H_2_O and NaCl (sat.); dried over Na_2_SO_4_, filtered and concentrated *in vacuo*. It afforded *tert*-butyl 3,4-dibromo-5-oxocyclooct-1-enecarboxylate 6 (43.00 mg, 91%) which crystallizes in Hex/EtOAc (1:1 *v*/*v*), mp 161–162 °C, IR νmax (neat): 2976 and 2930 (C—H), 1712 (C=O), 1449 (C=C), 1369, 1292 (C—O), 1253, 1159, 1127, 1110 (C—Br) cm^-1. 1^H NMR (400 MHz; CDCl_3_): δ 1.47 (9*H*, s, COOC(CH_3_)_3_); 2.00–3.02 (6*H*, m, H-6, H-7, H-8); 4.23 (1*H*, d, J 11.2, H-4); 5.01 (1*H*, dd, J 11.2 and 9.6, H-3); 6.80 (1*H*, d, J 9.6, H-2). ^13^C RMN (50 MHz; CDCl_3_): δ 27.1 (CH_2_, C-7); 27.8 (CH_2_, C-8); 28.2 (CH_3_*x* 3, COOC(CH_3_)_3_); 37.8 (CH_2_, C-6); 46.8 (CH); 60.4 (CH); 82.2 (C,COOC(CH_3_)_3_); 137.5 (CH, C-2); 138.1 (C, C-1); 164.7 (C, COOC(CH_3_)_3_); 202.0 (C, C-5). HRMS (ESI) m/*z* calcd.for C_13_H_18_Br_2_O_3_ [*M*+Na]:402.9515; found 402.9543; R—*X*.

### Refinement

The hydrogen atoms were positioned geometrically, with C—H distances constrained to 0.93 Å (aromatic CH), 0.96 Å (methyl CH3), 0.97 Å methylene CH2), 098 Å (methine CH) and refined in riding mode with *U*_iso_(H) = xUeq(C), where *x* = 1.5 for methyl H atoms and *x* = 1.2 for all other atoms.

### Crystal data

**Table:**

C_13_H_18_Br_2_O_3_	*F*(000) = 760
*M**_r_* = 382.09	*D*_x_ = 1.669 Mg m^−^^3^
Orthorhombic, *P**c**a*2_1_	Cu *K*α radiation, λ = 1.54178 Å
Hall symbol: P 2c -2ac	Cell parameters from 9578 reflections
*a* = 14.0658 (4) Å	θ = 4.6–66.5°
*b* = 9.5990 (3) Å	µ = 6.76 mm^−^^1^
*c* = 11.2657 (3) Å	*T* = 298 K
*V* = 1521.07 (8) Å^3^	Prismatic, colourless
*Z* = 4	0.24 × 0.14 × 0.10 mm

### Data collection

**Table:**

Bruker APEXII CCD area-detector diffractometer	2170 independent reflections
Radiation source: fine-focus sealed tube	2153 reflections with *I* > 2σ(*I*)
graphite	*R*_int_ = 0.048
phi and ω scans	θ_max_ = 66.5°, θ_min_ = 4.6°
Absorption correction: multi-scan (*SADABS*; Bruker, 2006)	*h* = −16→16
*T*_min_ = 0.370, *T*_max_ = 0.509	*k* = −11→11
10215 measured reflections	*l* = −10→13

### Refinement

**Table:**

Refinement on *F*^2^	Secondary atom site location: difference Fourier map
Least-squares matrix: full	Hydrogen site location: inferred from neighbouring sites
*R*[*F*^2^ > 2σ(*F*^2^)] = 0.029	H-atom parameters constrained
*wR*(*F*^2^) = 0.075	*w* = 1/[σ^2^(*F*_o_^2^) + (0.0448*P*)^2^ + 0.2923*P*] where *P* = (*F*_o_^2^ + 2*F*_c_^2^)/3
*S* = 1.09	(Δ/σ)_max_ = 0.001
2170 reflections	Δρ_max_ = 0.32 e Å^−^^3^
166 parameters	Δρ_min_ = −0.46 e Å^−^^3^
1 restraint	Absolute structure: Flack (1983), 803 Friedel pairs
Primary atom site location: structure-invariant direct methods	Flack parameter: 0.06 (3)

### Special details

**Table:**

Geometry. All e.s.d.'s (except the e.s.d. in the dihedral angle between two l.s. planes) are estimated using the full covariance matrix. The cell e.s.d.'s are taken into account individually in the estimation of e.s.d.'s in distances, angles and torsion angles; correlations between e.s.d.'s in cell parameters are only used when they are defined by crystal symmetry. An approximate (isotropic) treatment of cell e.s.d.'s is used for estimating e.s.d.'s involving l.s. planes.
Refinement. Refinement of *F*^2^ against ALL reflections. The weighted *R*-factor *wR* and goodness of fit *S* are based on *F*^2^, conventional *R*-factors *R* are based on *F*, with *F* set to zero for negative *F*^2^. The threshold expression of *F*^2^ > σ(*F*^2^) is used only for calculating *R*-factors(gt) *etc*. and is not relevant to the choice of reflections for refinement. *R*-factors based on *F*^2^ are statistically about twice as large as those based on *F*, and *R*- factors based on ALL data will be even larger.

### Fractional atomic coordinates and isotropic or equivalent isotropic displacement parameters (Å^2^)

**Table:**

	*x*	*y*	*z*	*U*_iso_*/*U*_eq_	
Br1	0.76354 (2)	0.96781 (4)	0.67951 (5)	0.04630 (14)	
Br2	0.94176 (3)	0.72748 (4)	0.77399 (5)	0.05922 (16)	
O1	0.9457 (2)	1.4186 (4)	1.0183 (4)	0.0671 (10)	
O2	0.79492 (19)	1.3427 (3)	0.9957 (3)	0.0429 (6)	
O3	0.9590 (2)	0.9175 (4)	1.0552 (3)	0.0605 (8)	
C1	0.9148 (3)	1.2311 (3)	0.8856 (4)	0.0368 (7)	
C2	0.8510 (2)	1.1346 (3)	0.8551 (3)	0.0359 (7)	
H2	0.7890	1.1450	0.8824	0.043*	
C3	0.8733 (2)	1.0108 (3)	0.7796 (4)	0.0350 (7)	
H3	0.9285	1.0313	0.7294	0.042*	
C4	0.8964 (3)	0.8890 (4)	0.8617 (3)	0.0403 (7)	
H4	0.8391	0.8632	0.9060	0.048*	
C5	0.9749 (3)	0.9286 (4)	0.9494 (4)	0.0403 (8)	
C6	1.0669 (3)	0.9841 (5)	0.9021 (4)	0.0481 (10)	
H6B	1.0700	0.9652	0.8176	0.058*	
H6A	1.1189	0.9347	0.9398	0.058*	
C7	1.0806 (3)	1.1419 (5)	0.9221 (5)	0.0503 (10)	
H7B	1.0685	1.1627	1.0050	0.060*	
H7A	1.1463	1.1655	0.9057	0.060*	
C8	1.0164 (3)	1.2335 (4)	0.8455 (4)	0.0455 (9)	
H8B	1.0396	1.3286	0.8479	0.055*	
H8A	1.0197	1.2018	0.7638	0.055*	
C9	0.8876 (3)	1.3426 (3)	0.9735 (3)	0.0407 (8)	
C10	0.7509 (3)	1.4418 (4)	1.0807 (5)	0.0488 (10)	
C11	0.7934 (4)	1.4216 (6)	1.2022 (4)	0.0701 (13)	
H11A	0.7917	1.3246	1.2227	0.105*	
H11B	0.7576	1.4740	1.2594	0.105*	
H11C	0.8581	1.4534	1.2020	0.105*	
C12	0.7647 (6)	1.5884 (5)	1.0332 (7)	0.086 (2)	
H12A	0.8314	1.6092	1.0295	0.130*	
H12B	0.7338	1.6537	1.0849	0.130*	
H12C	0.7377	1.5950	0.9552	0.130*	
C13	0.6482 (4)	1.3957 (6)	1.0791 (6)	0.0723 (14)	
H13A	0.6223	1.4091	1.0011	0.108*	
H13B	0.6125	1.4497	1.1353	0.108*	
H13C	0.6444	1.2988	1.1000	0.108*	

### Atomic displacement parameters (Å^2^)

**Table:**

	*U*^11^	*U*^22^	*U*^33^	*U*^12^	*U*^13^	*U*^23^
Br1	0.0408 (2)	0.0566 (2)	0.0415 (2)	−0.00053 (14)	−0.0054 (2)	−0.01174 (18)
Br2	0.0757 (3)	0.0393 (2)	0.0626 (3)	0.01023 (16)	−0.0059 (2)	−0.0133 (2)
O1	0.0599 (18)	0.0619 (19)	0.079 (3)	−0.0142 (15)	0.0064 (16)	−0.0333 (17)
O2	0.0436 (13)	0.0401 (12)	0.0451 (15)	0.0075 (11)	0.0017 (12)	−0.0121 (11)
O3	0.0719 (19)	0.0752 (19)	0.0343 (17)	−0.0037 (15)	−0.0071 (14)	0.0051 (16)
C1	0.0403 (17)	0.0328 (15)	0.0374 (19)	0.0002 (13)	0.0039 (17)	−0.0032 (14)
C2	0.0355 (16)	0.0375 (15)	0.0347 (18)	0.0076 (13)	0.0050 (14)	−0.0044 (14)
C3	0.0347 (17)	0.0374 (14)	0.0330 (17)	−0.0026 (13)	0.0021 (16)	−0.0025 (15)
C4	0.0439 (18)	0.0394 (15)	0.038 (2)	−0.0016 (14)	0.0021 (17)	−0.0007 (14)
C5	0.0402 (19)	0.0435 (16)	0.037 (2)	0.0070 (16)	−0.0026 (16)	−0.0026 (16)
C6	0.0339 (19)	0.057 (2)	0.054 (3)	0.0070 (16)	−0.0030 (17)	−0.0068 (19)
C7	0.0347 (17)	0.060 (2)	0.056 (3)	−0.0034 (17)	0.0025 (17)	−0.0164 (19)
C8	0.044 (2)	0.0453 (17)	0.047 (2)	−0.0076 (15)	0.0098 (17)	−0.0082 (15)
C9	0.050 (2)	0.0352 (15)	0.037 (2)	0.0011 (15)	0.0018 (16)	−0.0017 (14)
C10	0.066 (2)	0.0397 (17)	0.040 (2)	0.0135 (17)	0.009 (2)	−0.0088 (18)
C11	0.088 (3)	0.082 (3)	0.041 (3)	−0.001 (3)	0.006 (2)	−0.006 (2)
C12	0.138 (5)	0.038 (2)	0.083 (4)	0.026 (3)	0.038 (4)	0.002 (2)
C13	0.063 (3)	0.076 (3)	0.078 (4)	0.019 (2)	0.005 (3)	−0.023 (3)

### Geometric parameters (Å, °)

**Table:**

Br1—C3	1.956 (4)	C6—H6A	0.9700
Br2—C4	1.946 (4)	C7—C8	1.528 (7)
O1—C9	1.206 (5)	C7—H7B	0.9700
O2—C9	1.328 (5)	C7—H7A	0.9700
O2—C10	1.486 (5)	C8—H8B	0.9700
O3—C5	1.217 (5)	C8—H8A	0.9700
C1—C2	1.334 (5)	C10—C11	1.507 (7)
C1—C8	1.499 (5)	C10—C13	1.511 (7)
C1—C9	1.508 (5)	C10—C12	1.518 (7)
C2—C3	1.495 (5)	C11—H11A	0.9600
C2—H2	0.9300	C11—H11B	0.9600
C3—C4	1.526 (5)	C11—H11C	0.9600
C3—H3	0.9800	C12—H12A	0.9600
C4—C5	1.529 (6)	C12—H12B	0.9600
C4—H4	0.9800	C12—H12C	0.9600
C5—C6	1.498 (6)	C13—H13A	0.9600
C6—C7	1.543 (6)	C13—H13B	0.9600
C6—H6B	0.9700	C13—H13C	0.9600
			
C9—O2—C10	122.1 (3)	C1—C8—C7	112.6 (4)
C2—C1—C8	125.0 (3)	C1—C8—H8B	109.1
C2—C1—C9	119.5 (3)	C7—C8—H8B	109.1
C8—C1—C9	115.4 (3)	C1—C8—H8A	109.1
C1—C2—C3	123.9 (3)	C7—C8—H8A	109.1
C1—C2—H2	118.1	H8B—C8—H8A	107.8
C3—C2—H2	118.1	O1—C9—O2	125.9 (3)
C2—C3—C4	108.0 (3)	O1—C9—C1	122.2 (3)
C2—C3—Br1	109.3 (2)	O2—C9—C1	111.9 (3)
C4—C3—Br1	110.8 (2)	O2—C10—C11	109.7 (4)
C2—C3—H3	109.6	O2—C10—C13	101.7 (4)
C4—C3—H3	109.6	C11—C10—C13	110.7 (5)
Br1—C3—H3	109.6	O2—C10—C12	108.3 (4)
C3—C4—C5	110.8 (3)	C11—C10—C12	112.9 (5)
C3—C4—Br2	111.9 (3)	C13—C10—C12	113.0 (5)
C5—C4—Br2	106.8 (2)	C10—C11—H11A	109.5
C3—C4—H4	109.1	C10—C11—H11B	109.5
C5—C4—H4	109.1	H11A—C11—H11B	109.5
Br2—C4—H4	109.1	C10—C11—H11C	109.5
O3—C5—C6	122.5 (4)	H11A—C11—H11C	109.5
O3—C5—C4	118.6 (4)	H11B—C11—H11C	109.5
C6—C5—C4	118.9 (3)	C10—C12—H12A	109.5
C5—C6—C7	113.9 (3)	C10—C12—H12B	109.5
C5—C6—H6B	108.8	H12A—C12—H12B	109.5
C7—C6—H6B	108.8	C10—C12—H12C	109.5
C5—C6—H6A	108.8	H12A—C12—H12C	109.5
C7—C6—H6A	108.8	H12B—C12—H12C	109.5
H6B—C6—H6A	107.7	C10—C13—H13A	109.5
C8—C7—C6	114.1 (4)	C10—C13—H13B	109.5
C8—C7—H7B	108.7	H13A—C13—H13B	109.5
C6—C7—H7B	108.7	C10—C13—H13C	109.5
C8—C7—H7A	108.7	H13A—C13—H13C	109.5
C6—C7—H7A	108.7	H13B—C13—H13C	109.5
H7B—C7—H7A	107.6		

### Hydrogen-bond geometry (Å, °)

**Table:**

*D*—H···*A*	*D*—H	H···*A*	*D*···*A*	*D*—H···*A*
C3—H3···O3^i^	0.98	2.57	3.525 (5)	165.
C8—H8A···O3^i^	0.97	2.63	3.590 (6)	172.

Symmetry codes: (i) −*x*+2, −*y*+2, *z*−1/2.
